# A growing understanding of the role of muscarinic receptors in the molecular pathology and treatment of schizophrenia

**DOI:** 10.3389/fncel.2023.1124333

**Published:** 2023-02-22

**Authors:** Brian Dean, Geor Bakker, Hiroki R. Ueda, Andrew B. Tobin, Alastair Brown, Richard A. A. Kanaan

**Affiliations:** ^1^Synaptic Biology and Cognition Laboratory, The Florey Institute of Neuroscience and Mental Health, Parkville, VIC, Australia; ^2^Sosei Heptares, Cambridge, United Kingdom; ^3^Department of Systems Pharmacology, Graduate School of Medicine, The University of Tokyo, Tokyo, Japan; ^4^Laboratory for Synthetic Biology, RIKEN Center for Biosystems Dynamics Research, Osaka, Japan; ^5^Advanced Research Centre (ARC), School of Molecular Bioscience, University of Glasgow, Glasgow, United Kingdom; ^6^Department of Psychiatry, Austin Health, The University of Melbourne, Heidelberg, VIC, Australia

**Keywords:** muscarinic M1 receptor, muscarinic M4 receptor, schizophrenia, sub-group, neuroimaging, postmortem CNS, xanomeline, KarXT

## Abstract

Pre-clinical models, postmortem and neuroimaging studies all support a role for muscarinic receptors in the molecular pathology of schizophrenia. From these data it was proposed that activation of the muscarinic M1 and/or M4 receptor would reduce the severity of the symptoms of schizophrenia. This hypothesis is now supported by results from two clinical trials which indicate that activating central muscarinic M1 and M4 receptors can reduce the severity of positive, negative and cognitive symptoms of the disorder. This review will provide an update on a growing body of evidence that argues the muscarinic M1 and M4 receptors have critical roles in CNS functions that are dysregulated by the pathophysiology of schizophrenia. This realization has been made possible, in part, by the growing ability to visualize and quantify muscarinic M1 and M4 receptors in the human CNS using molecular neuroimaging. We will discuss how these advances have provided evidence to support the notion that there is a sub-group of patients within the syndrome of schizophrenia that have a unique molecular pathology driven by a marked loss of muscarinic M1 receptors. This review is timely, as drugs targeting muscarinic receptors approach clinical use for the treatment of schizophrenia and here we outline the background biology that supported development of such drugs to treat the disorder.

## 1. Introduction

The reports of a successful phase III trial (Lahucik, 2022) and a large phase II trial (Brannan et al., 2021), suggest a coformulation of xanomeline and trospium can reduce the symptoms of schizophrenia without causing unacceptable side-effects. The use of xanomeline, a partial agonist with preference for muscarinic M1 and M4 receptors (CHRM1/CHRM4)^1^ (Bymaster et al., 1997), to treat schizophrenia was underpinned by a hypothesis reflecting many lines of evidence arguing that activating CHRM1 and CHRM4 would be a mechanism to reduce the severity of the symptoms of the disorder (Bymaster et al., 1999; Dean et al., 2003). These exciting new developments around targeting CHRM1 and 4 to treat schizophrenia mean it is timely to review the role for CHRMs in the molecular pathology and treatment of the disorder. The notion of targeting CHRMs in the clinical management of schizophrenia is not new as pan-CHRM antagonists were used extensively to control the extrapyramidal side effects of first-generation antipsychotic drugs (Montastruc et al., 2018). With regards to the symptoms of schizophrenia, it is notable that treatment with high affinity CHRM antagonists to control extrapyramidal side effects worsened the cognitive deficits in people with the disorder (Tracy et al., 2001). These data show that improving the side effects of antipsychotic drugs are not necessarily linked to improving clinical outcomes for people with schizophrenia.

Recent progress in developing drugs that target CHRMs to treat schizophrenia has occurred because of an increasing understanding of the molecular pathology of the disorder that suggested therapeutically beneficial drugs needed to activate, not inhibit, CHRMs (Bymaster et al., 2002; Dean et al., 2003). Moreover, such drugs needed to target specific, not all, CHRMs so as to give different biochemical and behavioral outcomes that would be beneficial when used to treat schizophrenia; the development of such drugs has been comprehensively reviewed elsewhere (e.g. (Felder et al., 2018; van der Westhuizen et al., 2020; Foster et al., 2021; Johnson et al., 2022). Hence, this review will focus on the data supporting a role for the CHRM1 and CHRM4 in the molecular pathology of schizophrenia and the physiological processes that are affected by the changes in CHRM1 and CHRM4 signaling that would be postulated to be occurring in people with the disorder.

## 2. CHRM1 and CHRM4 receptor functions: Studies using receptor knockout mice

DNA sequencing technologies established that, in mammals, there were 5 genes encoding different CHRMs (Liao et al., 1989). These receptors mediate a variety of physiological outcomes because they regulate different signaling pathways and are differentially distributed across tissues and cell types (Felder, 1995). The family of CHRMs consists of 5 receptors (designated CHRM1, CHRM2, CHRM3, CHRM4 and CHRM5) with CHRM 1, 3 and 5 modulating the activity of signaling pathways that include phospholipases A2, C, and D, as well as tyrosine kinase and a novel class of voltage-insensitive calcium channels. By contrast, CHRM 2 and 4 were shown to modulate phospholipase A2 and adenylate cyclase activity. These findings were initially based on the availability of cloned receptors expressed in cell culture; by contrast, studies in intact animals were limited because of the absence of compounds that could activate or inhibit individual receptors in native tissue.

The distribution of CHRMs also plays a critical role in the outcome of each receptor being activated by acetylcholine. CHRM1 has a widespread distribution throughout the CNS but its levels are highest in cortical regions and the hippocampus, at times constituting more than 40% of total CHRMs (Flynn et al., 1995). Importantly, the CHRM1 is mainly located post-synaptically on excitatory neurons (Mrzljak et al., 1993) and, although present across the whole cortex, the CHRM1 is most prominent in cortical layers III and V/VI (Harrison et al., 1991; Scarr et al., 2018a). The CHRM1 is predominantly located on pyramidal neurons in the cortex (Zhang et al., 2002; Scarr et al., 2018a) and hippocampus (Scarr et al., 2016a). The CHRM2 is highly expressed in the nucleus basalis and occipital cortex and is present at lower levels in the hippocampus, caudate putamen and other cortical regions (Flynn et al., 1995). Significantly, in the cortex the CHRM2 is located pre- and post-synaptically (Mrzljak et al., 1993), the pre-synaptic CHRM2 acting as auto-receptors aiding in controlling the synthesis and release of acetylcholine (Zhang et al., 2002). By contrast, the post-synaptic CHRM2 is present on excitatory synapses (Mrzljak et al., 1998) with close to a third of cortical GABAergic neurons expressing CHRM2 (Disney and Aoki, 2008). The distribution of CHRM3 is similar to CHRM1 but the level of that receptor is much lower than CHRM1 and, at least in the rat, the CHRM3 is reported to be present on glia as well as cortical pyramidal neurons (Levey et al., 1994). Levels of CHRM4 are highest in the striatum but make up approximately 20% of the total CHRMs in the cortex and hippocampus (Flynn et al., 1995). In the striatum the CHRM4 is often associated with dopaminergic receptors (Weiner et al., 1990) and it is suggested the CHRM4 is critical in the mechanism by which acetylcholine modulates dopamine transmission in the striatum and / or nucleus accumbens (Threlfell and Cragg, 2011). The CHRM5 has only been detected at very low levels in hippocampus, substantia nigra, and ventral tegmental area (∼ 5% of total CHRMs) (Vilaro et al., 1990). One outcome of this differential distribution of CHRMs is that different receptors can differentially respond to acetylcholine to modulate the many effects of that neurotransmitter across the CNS (Perry et al., 1999).

A greater understanding of the role of individual CHRMs in the physiology of the whole organism was achieved following the development of mice that did not express the gene for one, or multiple, CHRMs (Wess, 2004). Here, because of the focus on schizophrenia, we will review the data pertinent to CHRM1 and CHRM4 as these are now implicated in the molecular pathology and treatment of the disorder.

### 2.1. CHRM1

Using mice that did not express functional CHRM1, it was shown that receptor had a role in activating the mitogen-activated protein kinase (MAPK) pathway, a pathway that has critical roles in synaptic plasticity and maintaining cognitive ability (Wess et al., 2007). Significantly, whilst several studies confirmed the presence of deficits in neural plasticity in the forebrain of the CHRM1^–/–^, initial behavioral studies did not show clear deficits in their cognitive abilities (Miyakawa et al., 2001). This was because the behaviors studied, such as matching-to-sample tasks, involved hippocampal learning that did not require CHRM1 signaling. It was subsequently shown that whilst CHRM1^–/–^ mice had very small changes in hippocampal long-term potentiation, they had significant deficits in tasks requiring non-matching-to-sample working memory and consolidation (Anagnostaras et al., 2003). These findings allowed the conclusion that the CHRM1 was critical in maintaining cognitive-related functions that required cortical engagement.

Ongoing studies of the cortical CHRM1 have shown that CHRM1 agonist-stimulated activation of mitogen-activated protein kinases (MAPK) is essentially abolished, and agonist-stimulated phosphatidyl inositol (PI) hydrolysis is reduced by >60%, in primary cortical cells from newborn CHRM1**^–^**^/^**^–^** mice (Hamilton and Nathanson, 2001). Going beyond well-defined CHRM signaling pathways, HEK293 cells were used to show CHRM1 signaling effects gene expression (von der Kammer et al., 2001). This effect of CHRM1 signaling has been further studied in the CHRM1**^–^**^/^**^–^** mouse where there are extensive changes in gene expression that would affect many aspects of cortical functioning (Supplementary Figure 1; Dean and Scarr, 2021). The challenge that remains is to identify which changes in these functions are responsible for the cortical-mediated behavioral changes in the CHRM1**^–^**^/^**^–^** mouse.

At the sub-cortical level, the CHRM1**^–^**^/^**^–^** mouse has a two-fold increase in levels of extracellular dopamine in the striatum (Gerber et al., 2001) which could be responsible for its pronounced increase in locomotor activity (Miyakawa et al., 2001). Further changes in behaviors and physiology in the CHRM1**^–^**^/^**^–^** mouse have highlighted the important role of that receptor outside the CNS, notably counteracting bronchoconstriction, possibly by releasing a relaxing agent stored in respiratory epithelia or pulmonary nerves (Struckmann et al., 2003). This is unlikely to be the only peripherally mediated CHRM1 effect given the receptor has been shown to be expressed by cells in the cardiovascular system (Saternos et al., 2018), the lung (Haddad et al., 1996), gastric mucosa, skin, melanocytes and immunocytes (Schledwitz et al., 2021). Hence, as it is increasingly recognized the molecular pathology of diseases of the human CNS extend into the periphery (Scarr et al., 2015; Lai et al., 2016b), it will be important to consider if CHRM1-mediated pathophysiology in disorders such as schizophrenia also extends beyond the CNS.

### 2.2. CHRM4

Findings using the CHRM4**^–^**^/^**^–^** mouse have emphasized the strong interactions of the CHRM4 and the glutamatergic systems as those mice had increased sensitivity to the disruption of pre-pulse inhibition (PPI) following N-methyl-D-aspartate (NMDAR) receptor blockade by phencyclidine (Felder et al., 2001). These data are significant because abnormal control of PPI has been suggested to be a useful biomarker for schizophrenia (Mena et al., 2016). In addition, CHRM4**^–^**^/^**^–^** mice have markedly increased basal dopamine levels in the nucleus accumbens, with both d-amphetamine and phencyclidine causing a greater increase in dopamine efflux compared to wild type mice (Tzavara et al., 2004). Finally, there was a small, but significant, increase in basal locomotor activity (a mouse behavior associated with increased dopaminergic activity) in the CHRM4**^–^**^/^**^–^** mouse (Gomeza et al., 1999). These data all support the notion that the absence of the CHRM4 leads to increased dopaminergic activity in the CNS which is also argued to be involved in the genesis of psychotic symptoms in people with schizophrenia (Carlsson, 1977). This is therefore a biological basis for the suggestion that specifically activating the CHRM4 would be a mechanism to reduce the psychotic symptoms of schizophrenia (Bymaster et al., 2002). This hypothesis has gained support from a recent trial that has reported that the CHRM4 positive allosteric modulator (PAM), emraclidine, reduces the severity of both acute psychotic and acute negative symptoms in people with schizophrenia (Krystal et al., 2022). Importantly, all receptor-specific CHRM PAMs increase the activity of a single CHRM by binding to an allosteric binding site to increase the responsiveness of the receptor to acetylcholine by increasing the probability that the receptor will be activated by the neurotransmitter (Yohn and Conn, 2018).

It has also been reported that the enhancement of acetylcholine efflux caused by the pan-CHRM antagonist, scopolamine, was markedly reduced in the CHRM4**^–^**^/^**^–^** mouse mid-brain (Tzavara et al., 2004). These data show that the CHRM4 has critical roles in controlling both dopaminergic and cholinergic activity in sub-cortical regions. This could be why there was an absence of scopolamine-mediated suppression of haloperidol-induced catalepsy in the CHRM4**^–^**^/^**^–^** mouse (Karasawa et al., 2003; Fink-Jensen et al., 2011). These data suggest that, in addition to having the potential for direct effects on levels of psychotic symptoms (Krystal et al., 2022), activation of the CHRM4 may also act to lessen some of the unwanted effects of the dopamine D2 receptor antagonist drugs that are the mainstay of treating psychosis.

Outside the CNS, there is now preliminary evidence to show expression of CHRM4 in peripheral tissue, including the gastric mucosa, small intestine, skin, melanocytes and immunocytes (Schledwitz et al., 2021). In this regard, studies using CHRM4**^–^**^/^**^–^** have shown CHRM4 has an important role in mediating skin keratinocyte adhesion (Nguyen et al., 2004) in a way that facilitates wound repair through keratinocyte migration and wound re-epithelialization (Chernyavsky et al., 2004).

Until the availability of receptor knockout mice, due to the absence of CHRM receptor-specific drugs, it was impossible to allocate changes in specific behaviors or biochemical pathways to an individual CHRM. Hence the data from the CHRM1**^–^**^/^**^–^** and CHRM4**^–^**^/^**^–^** gave the first real insight into the diverse impact CHRM1 and CHRM4 signaling had on CNS function at the molecular and behavioral level (Wess et al., 2006). However, the synthesis of drugs that can specifically target individual CHRMs (Conn et al., 2009) and the development of new technologies, such as optogenetics (Deisseroth et al., 2006), have allowed alternative approaches to understanding the functions of CHRMs in the CNS and in the periphery.

### 2.3. Muscarinic M1 and M4 receptor signaling

The power of newer approaches to studying the cholinergic system in the CNS is demonstrated by a study that used optogenetic stimulation to cause a transient increase in acetylcholine release in the striatum, which caused a long-lasting increase in excitability of medium spiny neurons that was associated with a hyperpolarizing shift of action potential thresholds and a decreased hyperpolarization amplitude (Lv et al., 2017). The outcome of this was an increase in probability of excitatory postsynaptic potential-action potential coupling. It was then shown that CHRM1 selective antagonist prevented the ability of a CHRM1 selective positive allosteric modulator to potentiate an acetylcholine-mediated persistent increase in medium spiny neuron intrinsic excitability. This combined use of optogenetics and receptor-selective and specific drugs has therefore revealed the importance of the CHRM1 in mediating the function of medium spiny neurons in the striatum.

Critical to understanding the role of CHRMs in CNS functioning is an understanding of how these receptors modulate other neurotransmitters systems. It was therefore significant that studies localizing RNA to cells in the primate CNS show that the CHRM1 is located on extrinsic glutamatergic and monoaminergic afferents as well as intrinsic GABAergic afferents (Alcantara et al., 2001). These data have now been expanded to suggest potential functional outcomes from interactions between the CHRM1 and the glutamatergic system in the CNS. Hence, it has been shown that hippocampal CHRM1 facilitates cognitive function by interplay with the AMPA receptor GluA1 subunit (Zhao et al., 2018). In addition, it has been shown that CHRM1 modulation of the AMPA GluA1 receptor sub-unit involves signaling through protein kinase C and Ras (Chen et al., 2020), possibly by differential phosphorylation of the AMPA GluA1 receptor sub-unit (Zhao et al., 2019b). This pathway may have some clinical significance, as it is suggested that through this process CHRM1 activation could rescue β-amyloid mediated cognitive impairment (Zhao et al., 2019a), is an important pathophysiological process associated with Alzheimer’s disease (Hardy and Higgins, 1992).

As is the case for interactions between the CHRM1 and glutamatergic systems, recent receptor-specific studies continue to argue for a role of the CHRM1 in modulating cognition. For example, modulating CHRM1 signaling, using the receptor specific drug VU0453595, has been shown to enhance the performance of mice in cue-dependent water-based T-maze, a dorsolateral striatum-dependent learning task (Lv et al., 2017). However, this may not be a simple linear relationship, as it has been reported that overstimulation of the CHRM1 in the primate prefrontal cortex can disrupt working memory (Vijayraghavan et al., 2018). In the primate cortex, and in rodent cortex, CHRM1 is known to modulate working memory *via* multiple pathways that include the N-methyl-D-aspartate receptor (Galvin et al., 2021) and KCNQ potassium channels (Galvin et al., 2020). It would therefore be worthwhile to determine if any or all of these mechanisms become non-responsive after overstimulation of the CHRM1.

Whilst studies focused on the CHRM4 may be less numerous, it has been reported that activating that receptor modulates both acquisition and consolidation of memory functions (Gould et al., 2018). In addition, striatal and nigral CHRM4 signaling leads to multilevel inhibition of striato-nigral pathways which can result in an attenuation of dyskinesia (Brugnoli et al., 2020), which links the CHRM4 to dopaminergic activity in the CNS (Porras et al., 2014). In addition, it has recently been shown that the CHRM4 in the striatum, thalamus and intergeniculate leaflet causes changes to the biological rhythm of locomotor activity in mice (Riljak et al., 2020).

### 2.4. Muscarinic receptors and REM and non-REM sleep

It has long been known that multiple neurotransmitter systems are involved in regulating REM sleep (Fraigne et al., 2015; Falup-Pecurariu et al., 2021) with cholinergic neurotransmission being important in controlling REM sleep (Yamada and Ueda, 2020). Due to the focus of our review we will provide commentary on a growing understanding of the role of CHRM1 in regulating REM sleep because a broader review of the role of the cholinergic system in REM sleep is available elsewhere (Yamada and Ueda, 2020).

Like many aspects of understanding the mechanisms by which acetylcholine can influence physiological outcomes, understanding how acetylcholine influences sleep has been hampered by the absence of appropriate experimental tools. However, with the development of technologies such as triple-CRISPR and ES-mice (Sunagawa et al., 2016; Ukai et al., 2017) it is now possible to understand the mechanisms by which acetylcholine can control sleep. Hence, it has now been shown that the CHRM1**^–^**^/^**^–^** / CHRM3**^–^**^/^**^–^** double knockout mouse has an enrichment of EEG theta oscillation during sleep, leaving the theta oscillation largely unaffected during wakefulness (Niwa et al., 2018). This study advanced earlier findings that showed pan-muscarinic antagonists such as atropine or scopolamine reduce the theta oscillation in anesthetized animals (Kim and Jeong, 1999; Buzsaki, 2002) to suggest the CHRM1 and CHRM3 are critically involved in controlling REM. Moreover, extensive use of newer gene modification techniques has shown that nicotinic acetylcholine receptors failed to affect sleep phenotypes (Niwa et al., 2018). In addition, it has been shown that CHRM1**^–^**^/^**^–^** mice have shorter REM sleep duration, but only slightly shorter NREM sleep duration, whilst CHRM3**^–^**^/^**^–^** mice have shorter NREM sleep duration, but their REM sleep duration was comparable to that of WT mice (Niwa et al., 2018). These findings suggest further studies are needed to better understand the molecular and cellular mechanisms involved in these receptor specific phenotypes.

Without direct experimental evidence, it is of interest that CHRM1 and CHRM3 make up roughly 60 and 10% of the total muscarinic acetylcholine receptors in the hippocampus (Dasari and Gulledge, 2011), respectively. This could be important because slow-wave and delta oscillations originate from the neocortex and the thalamus, respectively, whereas theta oscillation is thought to originate from the hippocampus (Sirota et al., 2008). Hence the differential distribution of CHRM1 and CHRM3 across the regions and different cells in the hippocampus may be critical to their effect on sleep. However, before these data are available it will be of great interest to see if drugs such as KarXT, when used to treat schizophrenia, may improve the sleep disturbances associated with the disorder. Such an outcome would seem possible given the recent report that the CHRM1 positive allosteric modulator, VU0453595, has been shown to modulate arousal and sleep / wake architecture in young and aged rodents, and nonhuman primates (Gould et al., 2020).

### 2.5. Muscarinic receptors and schizophrenia: Postmortem CNS studies

The first evidence suggesting there were changes in levels of CHRMs in people with schizophrenia was generated using postmortem CNS and radioligand binding. Using such an approach, it was reported that levels of [^3^H]pirenzepine binding were lower in the striatum (Dean and Crook, 1996), dorsolateral prefrontal cortex (DLPFC) (Crook et al., 2001), the anterior (Zavitsanou et al., 2004) and posterior cingulate cortex (Newell et al., 2007), superior temporal gyrus (Deng and Huang, 2005), premotor cortex (Dean et al., 2008b) and hippocampus (Crook et al., 2000) compared to non-psychiatric controls. Notably, compared to controls, [^3^H]pirenzepine binding was not altered in the thalamus of people with schizophrenia (Dean et al., 2004). In interpreting these results, it is important to note the various components that contribute to the selectivity of any radioligand binding assay. In the case of our radioligand binding assay it was important that the use of cloned CHRMs showed that unlabeled pirenzepine bound with high affinity to the CHRM1 and with a slightly higher affinity than it did to other CHRMs as the drug had 6, 7, 13 and 17 fold lower affinity for CHRM4, CHRM5, CHRM3 and CHRM2, respectively (Buckley et al., 1989). These data indicate the use of pirenzepine gives any assay a slight selectivity for the CHRM1. However, the overall selectivity of a radioligand binding assay depends on the selective binding of the radioligand and the displacing agent used to define non-specific binding (Bylund and Toews, 1993). In developing our radioligand binding assay we used unlabeled drugs that were selective for CHRM1 such as quinuclidinyl xanthene-9 -carboxylate hemioxilate (Crook, 1998) or 3-quinuclidinyl benzilate (Stanton et al., 1993) to increase selectivity for that receptor. Finally, we showed that the on-rate of [^3^H]pirenzepine binding for the CHRM1, as demonstrated by the binding of that radioligand to human cloned CHRM1 and CHRM4 (Scarr and Dean, 2008), was considerably faster than for the CHRM4 which means that measuring [^3^H]pirenzepine binding after 30 min incubations results in the radioligand binding being highly selective for the CHRM1. The selectivity of binding under these conditions have been confirmed using tissue from mice lacking CHRM1 or CHRM4 (Scarr and Dean, 2008; Gibbons et al., 2013), components of snake venom that show strong selectivity for the CHRM4 and CHRM4 specific allosteric modulators (Valuskova et al., 2018). Hence, the studies showing lower levels of [^3^H]pirenzepine binding in people with schizophrenia where the incubation time was short (Dean et al., 1996, 2008b; Crook et al., 2000, 2001) argue the decreased radioligand binding would be due to lower levels of CHRM1. By contrast, decreased [^3^H]pirenzepine binding in the studies using a longer incubation (Zavitsanou et al., 2004; Deng and Huang, 2005; Newell et al., 2007) could be due to lower levels of either receptor in the CNS of people with schizophrenia.

It is of significance that lower levels of cortical [^3^H]pirenzepine binding in schizophrenia shows some diagnostic specificity. This is because, compared to controls, cortical [^3^H]pirenzepine binding was not different in the cortex of people with major depressive disorders (Zavitsanou et al., 2004; Gibbons et al., 2009), bipolar disorders, and Alzheimer’s disease (Scarr et al., 2017). In addition, cortical [^3^H]pirenzepine binding has been reported as unaltered (Mcomish et al., 2017) or increased (Lange et al., 1993) in Parkinson’s disease.

Understanding the mechanisms causing lower levels of cortical [^3^H]pirenzepine binding has been advanced by studies showing lower levels of CHRM1 gene expression (Dean et al., 2002; Mancama et al., 2003) and protein in the DFPLC of people with schizophrenia. By contrast, it has been reported that levels of CHRM2, CHRM3 (Scarr et al., 2006) and CHRM4 (Dean et al., 2002) were not altered in the DFPLC from those with the disorder. In addition, changes in CHRM1 (Salah-Uddin et al., 2009), but not CHRM2/4, signaling has been reported in the DFPLC in people with schizophrenia.

Hence, current data would argue that changes in CHRM1 in the cortex, but not necessarily other CNS regions, are a significant component of the molecular pathology of schizophrenia.

### 2.6. The CHRM1 and the syndrome of schizophrenia

Schizophrenia is recognized as being a syndrome of disorders which present with the same defining constellation of symptoms (Jablensky, 2010) and it is now recognized that dissecting the syndrome into more homogeneous sub-groups is critical to advancing understanding of the pathophysiology of the syndrome and its components (Jablensky, 2006; Tamminga et al., 2017). Hence, it is significant that approximately 25% of people with schizophrenia could be separated into a sub-group, termed the CHRM deficit sub-group (MRDS), because they have a marked loss of cortical CHRM1 (Scarr et al., 2009).

The identification of MRDS has enabled studies to compare its molecular pathology to schizophrenia where there is no determinable loss of cortical CHRM1 (non-MRDS). This approach has shown that those with MRDS have no increased association with any sequence variation within the CHRM1 gene or its promotor (Scarr et al., 2009), exposure to antipsychotic drugs (Dean et al., 2015, 2016a, 2008b; Scarr et al., 2018a,c), the anticholinergic load in the cortex (Dean et al., 2020) or smoking status in MRDS compared to non-MRDS. By contrast, a number of changes in biochemical homeostasis comparing MRDS and non-MRDS have been identified (Table 1). For example, there are differential changes in gene expression (Scarr et al., 2018c) and levels of micro-RNAs controlling CHRM1 gene expression and translation (Scarr et al., 2013a) in MRDS compared to non-MRDS. By contrast, changes in levels of transcription factors regulating CHRM1 expression did not differ between MRDS and non-MRDS (Seo et al., 2014; Scarr et al., 2018c). This proposed mechanism is supported by data showing that, compared to controls and people with non-MRDS, those with MRDS do not have changes in innervating cholinergic neurons as indicated by levels of choline acetyltransferase (Dean et al., 2020) but have lower levels of CHRM1 in many cortical areas (Gibbons et al., 2013; Seo et al., 2014), lower responsiveness to CHRM1 allosteric modulation (Dean et al., 2016a; Hopper et al., 2019), and higher levels of the α7 nicotinic receptor (Dean et al., 2020; Table 1). The higher levels of α7 nicotinic receptor in people with MRDS argues that the sub-group may have a breakdown in cholinergic homeostasis that is not limited to the CHRM1 and CHRM4, and that MRDS may be particularly responsive to being treated with drugs that can selectively target the α7 nicotinic receptor [as argued by Jones et al. (2012)] and the CHRM1.

**TABLE 1:** A summary of differential findings relating to the molecular pathology of a sub-group of people with schizophrenia and a marked loss of cortical CHRM1 (MRDS) and other people with schizophrenia who do not have a marked loss of cortical CHRM1 (non-MRDS) compared to controls.

	MRDS	Non-MRDS
Dorsolateral prefrontal cortex	Markedly lower level of CHRM1 in multiple cortical regions.	
	Unique changes in gene expression in the dorsolateral prefrontal cortex.	
	Higher levels of α7 nicotinic receptors in the dorsolateral prefrontal cortex.	
	Changed CHRM1 promotor methylation.	
	Higher levels of the microRNA 107.	
	Lower numbers of CHRM1 positive neurons in laminae III and IV and lower levels of CHRM1 in the dorsolateral prefrontal cortex.	Lower number of CHRM1 positive neurons in laminae III and IV with unchanged levels of CHRM1 in the dorsolateral prefrontal cortex.
	Unchanged level of innervating cholinergic neurons in the dorsolateral prefrontal cortex.	Unchanged level of innervating cholinergic neurons in the dorsolateral prefrontal cortex.
	Higher level of RNA encoding the zinc transporter SLC39A12.	Higher level of RNA encoding the zinc transporter SLC39A12.
	Higher level of RNA encoding selenium binding protein.	Higher level of RNA encoding selenium binding protein.
	Lower level of RNA encoding phospholipase C beta 1.	Lower level of RNA encoding phospholipase C beta 1.
Hippocampus	Markedly lower level of CHRM1.	
Striatum	Markedly lower level of CHRM1.	
		Changes in pyruvate kinase, pyruvate and lactate consistent with an inability to process glucose through the Embden-Meyerhof-Parnas pathway.

The notion of sub-group specific changes in CHRM1-related biochemistry has recently been suggested to account for changes in the coupling of CHRM1 to Gα _*q/11*_ in the dorsolateral prefrontal cortex of people with schizophrenia (Odagaki et al., 2020). This suggestion adds to an earlier report showing that abnormalities in CHRM1-coupling to Gα _*q/11*_ was altered in people with MRDS but not in non-MRDS (Salah-Uddin et al., 2009). By contrast, using tissue from the same cases, CHRM1-coupling to Gα _*q/11*_ was not altered in MRDS or non-MRDS when the receptor was activated using the selective CHRM1 allosteric agonist AC-42 (Salah-Uddin et al., 2009). Notably, it is recognized that the CHRM1 can undergo conformational changes depending on the receptor occupancy state at different sites on the receptor (Maeda et al., 2019). It is therefore intriguing to postulate whether the orthosteric binding signaling in schizophrenia and MRDS could be due to some intrinsic modulator of receptor binding as has been suggested for the GABA_*A*_ receptor (van Kammen et al., 1993) and the serotonin 2A receptor (Dean et al., 2008a).

When considering the breakdown in cholinergic homeostasis in schizophrenia it is significant that people with MRDS and non-MRDS have lower levels of CHRM1-expressing pyramidal cells in laminae III and V of the DLPFC (Scarr et al., 2018a). Notably, this loss of CHRM1-positive pyramidal cells occurred without any changes in total neuron and glia cell number (Scarr et al., 2018a) or in changes in levels of pre-synaptic boutons as measured using synaptosome nerve-associated protein 25 (Dean et al., 2020). These data strongly argue there must be aberrant CHRM1 signaling in both MRDS and non-MRDS because fewer pyramidal cells will respond to stimulation by acetylcholine. There are also other biochemical changes common to both MRDS and non-MRDS, which include higher levels of cortical RNA encoding the zinc transporter SLC39A12 (Scarr et al., 2016b), selenium binding protein (Udawela et al., 2015) and lower levels of phospholipase C beta 1 RNA (Udawela et al., 2017). In addition, people with non-MRDS have been shown to have changes in levels of pyruvate kinase in the striatum (Table 1), an enzyme which is critically linked to the activity of the Embden-Meyerhof pathway (Dean et al., 2016b). These data are of interest because neuroimaging studies identified a sub-group of patients with schizophrenia that had abnormalities in striatal glucose utilization (Buchsbaum et al., 1992); it is intriguing to postulate whether this was due to abnormal glucose utilization due to the altered levels of pyruvate kinase observe in non-MRDS.

Whilst there is a growing understanding of disturbances in cortical homeostasis in MRDS and non-MRDS, recent data shows there are lower levels of CHRM1 in the hippocampus and striatum in those in the MRDS sub-group (Dean et al., 2016a; Hopper et al., 2019). Hence further studies comparing changes in hippocampal and sub-cortical molecular pathology will need to be conducted to fully understand the pathophysiology of MRDS.

Whilst it has long been argued that CHRM1 plays an important role in the pathology of schizophrenia (Raedler et al., 2007), recent findings suggest the receptor is important in regulating diverse functions that are affected in those with the disorder. For example, the CHRM1 and CHRM3 have been shown to be important in odor discrimination and learning (Chan et al., 2017), a deficit that has been demonstrated in people with schizophrenia (Brewer et al., 2001). It has also been shown that Δ^9^-tetrahydrocannabinol, given to rats during adolescence, decreases the expression and function of cortical CHRM1 (Garzon et al., 2021). This is significant given the relatively high use of cannabis by people with schizophrenia (Winklbaur et al., 2006). People with schizophrenia are known to have an increased risk of sleep disturbance (Kaskie et al., 2017). Importantly, sleep is a cyclic process that is composed of rapid eye movement (REM) and non-rapid eye movement (NREM) sleep (Pocivavsek and Rowland, 2017). In schizophrenia, a number of studies have reported reductions in the total time sleeping and a greater latency to fall asleep, decreases in REM latency and duration as well as reductions in total NREM (Pocivavsek and Rowland, 2017). Pertinent to this review, it is known that acetylcholine is crucial in controlling REM sleep (Hobson et al., 1975; Sakai et al., 2001).

### 2.7. CHRM4 and the molecular pathology of schizophrenia

Current data suggests that levels of CHRM4 are not altered in the prefrontal cortex or parietal cortex of people with schizophrenia (Dean et al., 2002). However, the neocortex contains diverse and separate regions in which biochemical homeostasis is differentially affected by the molecular pathology of the disorder (Scarr et al., 2018b). It therefore remains possible that changes in cortical CHRM4 levels have yet to be identified within some cortical regions. It is also noteworthy that there are decreased levels of [^3^H]pirenzepine binding across many regions of the hippocampus (Crook et al., 2000; Scarr et al., 2007). One of these studies also reported lower levels CHRM4, but not CHRM1, mRNA across the hippocampus from people with schizophrenia compared to controls (Scarr et al., 2007). These data would argue that at least some of the decrease in [^3^H]pirenzepine binding in schizophrenia was due to low levels of CHRM4. In the striatum, it has been reported that levels of [^3^H]pirenzepine binding (Dean et al., 1996) but not CHRM1 or CHRM2 mRNA (Dean et al., 2000), were lower in people with schizophrenia compared to controls. These data would favor the argument that lower levels of CHRM4 were contributing to the lower levels of [^3^H]pirenzepine binding in the striatum of people with schizophrenia. However, it is clear more studies on CHRMs in the hippocampus and striatum are warranted to get a clear understanding about the changes in those receptors in schizophrenia.

### 2.8. Genetics, muscarinic receptors, and cognition in schizophrenia

It is now widely accepted that schizophrenia occurs in individuals with a genetic susceptibility after adverse environmental exposure (Smigielski et al., 2020). With regards to CHRM1 levels in people with schizophrenia, current data suggests that the lower levels of CHRM1 are not associated with a gene variant (Scarr et al., 2009) but could be due to changes in gene methylation (Scarr et al., 2013a) which is affected by environmental factors (Smigielski et al., 2020). However, based on findings using DNA from blood, there may be an association between genotype at a 267C/C single nucleotide polymorphism in the *CHRM1* gene and cognitive functioning in schizophrenia (Scarr et al., 2012).

As well as a direct link between the *CHRM1* gene sequence and cognition, it has also been reported that low levels of cortical CHRM1 are associated with single nucleotide polymorphisms in the catechol-*O*-methyltransferase (*COMT*) gene that are associated with poorer cognitive ability (Dean and Scarr, 2016). These data would suggest that people with schizophrenia who have lower levels of CHRM1 could, as has previously been suggested (Egan et al., 2001), have cognitive deficits that are at least in part due to a breakdown in homeostasis in pathways regulated by COMT. Initially it was postulated that the linked between COMT genotype at rs4680 (valine to methionine substitution: Val 108/158 Met) and cognition was due to the methionine substitute COMT being less able to metabolize dopamine leading to an increase in the storage and release of dopamine from the pre-synaptic neuron (Weinshilboum et al., 1999). This increased release of dopamine was important because of evidence to suggest that increased dopaminergic activity in the prefrontal cortex was linked to levels of working memory (Goldman-Rakic, 1997). However, it has been shown that COMT can affect the metabolism of both dopamine and noradrenaline (Eisenhofer and Finberg, 1994) and noradrenaline has also been shown to modulate cognitive functioning (Holland et al., 2021). Hence it is likely that COMT could affect cognition by affecting levels of both dopamine and noradrenaline. Significantly the association between COMT genotype and levels of CHRM1 was linked to two genotypes (rs4680 and rs4818) (Dean and Scarr, 2016). This is significant because rs4818 is linked to cognitive ability in humans (Roussos et al., 2008) but this association does not primarily involve the modulation of the catecholaminergic system, rather it is due to and alteration in the structure of COMT RNA which results in altered levels of translation of the RNA to protein (Nackley et al., 2006). This mechanism, as well as the modulation in catecholamine activity, could be modulating the link between COMT genotype and cognition in humans. This mechanism is of interest because it has been reported that that genotype at rs4680 and rs4818 are associated with differing levels of soluble COMT (S-COMT), but not membrane-bound COMT (MB-COMT), in the human cortex (Parkin et al., 2018). This finding is relevant because MBCOMT, which is localized on the cell membrane and present on cell bodies, axons and dendrites of neurons, has a role in the breakdown of the catecholamines (Chen et al., 2011) but S-COMT is localized in the cytoplasm where it has access to many more catechol-based substrates that include the catechol estrogens (Schutze et al., 1994). This means that S-COMT can impact on cellular functioning by altering levels of catechol estrogens which will affect the occupancy and nuclear localization of estrogen receptors (Schutze et al., 1994; Levin, 2001) and thereby alter levels of the expression of genes that have estrogen response elements (Klinge, 2001). Hence, the link between levels of CHRM1 expression and COMT genotypes likely involves the actions of S-COMT *via* its ability to indirectly modulate gene expression which could be either an alternative or cooperative pathway to the ability of M-COMT to modulate the functioning of dopamine and noradrenaline (Parkin et al., 2018).

There may also be a role for the CHRM4 gene as a susceptibility gene for schizophrenia, as it has been reported that the C/C allele at the 1341 single nucleotide polymorphism carries an increased risk for schizophrenia that is resistant to treatment with current drugs (Scarr et al., 2013b). More recently, it has been suggested that the T/T allele at the same single nucleotide polymorphism was associated with an increased risk of developing schizophrenia (Pozhidaev et al., 2020). It is not readily apparent why such different outcomes could occur, but there are significant methodological differences between the studies that includes using cohorts with different genetic backgrounds. This could be important as a study comparing cortical [^3^H]pirenzepine-binding in Japanese people with schizophrenia to controls from Australia failed to show any differences between the diagnostic cohorts (Matsumoto et al., 2005).

Despite these potentially interesting findings, it must be acknowledged that no strong association between CHRMs gene sequence variation and schizophrenia has been established in the growing large GWAS database (Dennison et al., 2020).

### 2.9. Muscarinic receptor molecular imaging studies in schizophrenia

Studies investigating levels of CHRMs and their relationship to CNS function in people with schizophrenia have been limited by a lack of highly selective radioligands and because such studies would preferably involve medication-free people with the disorder. However, given the difficulties in studying CHRMs using medication-free people with schizophrenia it has been deemed worthwhile to study people with schizophrenia who are not being treated with anticholinergic drugs to control extrapyramidal-side effects (Caradoc-Davies et al., 1986) or with atypical antipsychotic drugs, such as olanzapine, that occupy CHRMs (Bymaster et al., 2003).

**FIGURE 1:**
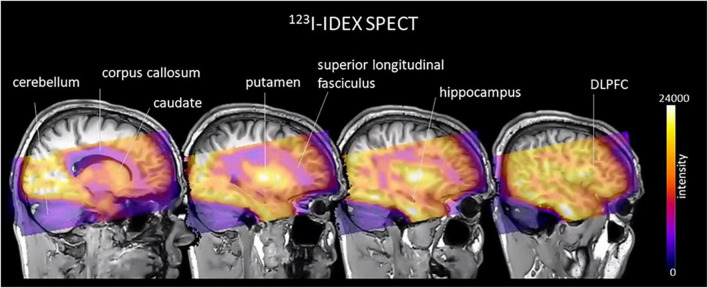
An example of an image, in cool-warm colors, obtained after ^123^I-IDEX SPECT scan registered and overlain onto T1 weighted anatomical MRI scan showing high specific preferential binding to CHRM1 and CHRM4 receptors in the cortex, including the dorsolateral prefrontal cortex (DLPFC), hippocampus, and striatum, with no uptake in the cerebellum, which is devoid of CHRM1 and CHRM4.

Molecular imaging studies measuring CHRMs in people with schizophrenia have been possible using two non-selective single photon emission compute tomography (SPECT) tracers 3-quinuclidinyl-4-[^123^I] iodobenzilate (^123^I-QNB) and N-methyl-4-[^123^I] iododexetimide (^123^I-IDEX) that have high affinities for both the CHRM1 and CHRM4 (Muller-Gartner et al., 1992; Boundy et al., 1995; Piggott et al., 2002). The distribution of both ^123^I-IQNB and ^123^I-IDEX reflect the distribution of CHRM1 and CHRM4 in the human CNS showing high levels of uptake in the cortex, hippocampus, and striatum (Figure 1). Importantly, both tracers are antagonists and therefore their signal is not specific to functional receptors in the high affinity state. The pharmacological characterization of ^123^I-IDEX and ^123^I-IQNB showed ^123^I-IDEX was a more selective CHRM1/CHRM4 tracer that has a higher affinity for CHRM1 (picomolar range) over CHRM4 (nanomolar range) and was preferentially displaced by CHRM1 agonists and antagonists (Bakker et al., 2015). Recognizing the limitations of current ligands, there are ongoing efforts to identify agonist positron emission tomography (PET) tracers that are able to differentiate between CHRM1 and CHRM4 (Table 2; Ozenil et al., 2021). In addition, a fluorinated PET version of dexetimide has been synthesized (Rowe et al., 2021) which has been reported to be safe to use in humans and has a CNS distribution consistent with the ligand targeting CHRM1 and CHRM4.

**TABLE 2:** Overview of radioligands used to evaluate M_1_ and M_4_ receptor binding in schizophrenia and promising selective positron emission tomography (PET) radioligands for future research to understand functional changes in M_1_ and M_4_ receptors.

Radioligand	[^123^] I-IQNB	[^123^] I-IDEX	[^11^]C-LSN31721765	[^11^]C-MK-6884
Radioligand structure	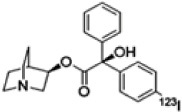	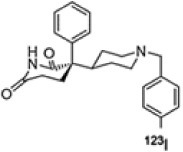	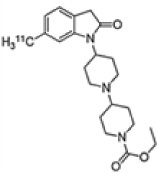	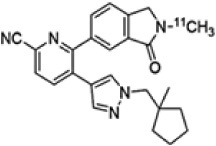
Imaging modality	SPECT	SPECT	PET	PET
Spacial resolution	Low	Low	High	High
Mode of action	Antagonist	Antagonist	Agonist (bitopic ligand)	Positive allosteric modulator
Specificity	Non-selective CHRM1/M4	Non-selective CHRM1 > CHRM4	CHRM1 selective	CHRM4 selective
Signal interpretation	CHRM1 + CHRM4	CHRM1 + CHRM4 but CHRM1 in CHRM1 rich CNS regions	CHRM1	Basal binding can reflect basal cholinergic tone. Simple tasks can be used to activate areas of interest to reflect CHRM4 function
Reflect high affinity state/g-coupled state	No	No	Yes	Yes
Published data	Schizophrenia neurodegenerative disorders	Psychotic disorders neurodegenerative disorders	Healthy volunteers only	Alzheimer’s disease

The need for neuroimaging ligands has become critical to study CHRM target engagement because of recently reported clinical trials in people with schizophrenia. These trials included phase II and III trials showing the efficacy of KarXT, which is predicted to having its therapeutic benefits from the inclusion of CHRM1/CHRM4 dual agonism, against the positive and negative symptoms of schizophrenia (Brannan et al., 2021). In addition, the recent report that emraclidine, a specific CHRM4 PAM, lessons psychotic and negative symptoms in people with schizophrenia (Krystal et al., 2022).

The initial neuroimaging study of CHRMs in schizophrenia used ^123^I-IQNB SPECT to show there were lower levels of CHRM1/CHRM4 (30% in the striatum and 20% in frontal and temporal regions) in the CNS of drug-free people with the disorder compared to matched control subjects (Raedler et al., 2003). Notably, lower levels of ^123^I-IQNB binding were associated with worse positive symptom severity. This study added to an earlier study using ^123^I-IDEX SPECT that reported lower levels of CHRM1/CHRM4 in people with schizophrenia who were being treated with risperidone (Lavalaye et al., 2001). Importantly, in contrast to some other atypical antipsychotics, risperidone does not occupy CHRMs and therefore would not affect ^123^I-IDEX binding (Lavalaye et al., 2001; Corena-Mcleod, 2015). In this study, those with the schizophrenia had approximately a 2.5-fold lower level of CHRM1/CHRM4 in the frontal, striatal, temporal, and occipital cortex compared to controls (Lavalaye et al., 2001). In interpreting the two studies, the study showing lower ^123^I-IDEX would be preferentially reflecting lower levels of CHRM1, except for the striatum, where CHRM1 levels are lower and the binding of the radioligand would reflect levels of CHRM1 and CHRM4. Although both studies support lower levels of CHRM1/CHRM4 in people with schizophrenia, neither of the studies were sufficiently powered to identify the MRDS subgroup identified using postmortem CNS (Scarr et al., 2009). Moreover, neither study quantified CHRM1/CHRM4 in the hippocampus, a region important in modulating learning and memory which have shown impairments in people with schizophrenia (Lodge and Grace, 2011).

More recent research has attempted to address some of the limitations of the early studies on CHRMs in schizophrenia by measuring CHRM1/CHRM4 using SPECT and ^123^I-IDEX binding in 30 medication-free people who were diagnosed with a psychotic disorder and were in the early phase of the disease (Bakker et al., 2018). In this study there was no evidence for the subgroup of people with extremely low levels of CHRM1/CHRM4 that defined MRDS (Scarr et al., 2009). This could be because the study included people with a range of psychotic disorders, which raises the possibility that MRDS may be specific to schizophrenia and would therefore be less apparent in a mixed group. Importantly, this study did show a significant relationship between lower ^123^I-IDEX binding in the DLPFC and more severe negative symptoms, supporting a more prominent role for the CHRM1 in the genesis of those symptoms. This finding is in line with improvements in negative symptoms in a subset of people after treatment with clozapine which were suggested to be due to the clozapine metabolite n-desmethylclozapine acting as a CHRM1 agonist (Meltzer, 1992, 1997; Costa-Dookhan et al., 2021). Despite these encouraging results no data suggests that treatment with n-desmethylclozapine as a monotherapy is beneficial in the treatment of schizophrenia (Mendoza and Lindenmayer, 2009).

Another finding from the study of ^123^I-IDEX to CHRM1/CHRM4 binding in psychosis was that lower levels of binding in the DLPFC was related to worse verbal learning and memory scores whereas lower hippocampal binding predicted worse delayed recall (Bakker et al., 2018). These findings were further investigated in the same patients using functional magnetic resonance imaging and a task measuring functional activation during visual spatial learning and memory after treatment with biperiden (Bakker et al., 2020). Importantly, biperiden is a selective CHRM1/CHRM4 antagonist (10-fold more selective for CHRM1 over CHRM4) that is known to induce significant deficits in learning and memory in both patients with schizophrenia and controls (Vingerhoets et al., 2017). The outcome of the study with biperiden was the demonstration of a significantly greater activation in response to biperiden in the parahippocampal and superior temporal gyrus in the people with psychosis during both learning and memory tasks compared to controls (Bakker et al., 2020). In addition, lower CHRM1/CHRM4 binding in the hippocampus during learning and CHRM1 binding in the DLPFC during memory, predicted a greater magnitude of abnormal hyperactivity (Bakker et al., 2020). These data argue that there may be a loss of CHRM1/CHRM4 reserve in people with psychotic disorders early in the onset of the disorder that is giving an increased sensitivity to biperiden.

Taken together, data from postmortem CNS show there are lower levels of CHRM1/CHRM4 in some people with schizophrenia. Given the lack of agonist SPECT or PET tracers, current studies provide no direct evidence as to whether there is a loss of receptors in the high affinity (g-coupled) state in people with schizophrenia, but this limitation may be overcome with the development of 11C-MK6884 (CHRM4 PAM) and 11C -LSN3172176 (bitopic CHRM1 agonist) PET tracers.

### 2.10. Health comorbidities and schizophrenia

There are now data that argue that the molecular pathology of schizophrenia extends beyond the CNS (Glatt et al., 2005; Scarr et al., 2015; Fillman et al., 2016; Lai et al., 2016a; Dean et al., 2022) and the peripheral effects of xanomeline (Shekhar et al., 2008) was a problem in taking the drug into the clinic. Thus, although the major focus on understanding schizophrenia has been on the perturbations in CNS function and potential mechanisms that could underlie the genesis of the constellation of symptoms needed to make a diagnoses (American Psychiatric Association, 2013) it is now argued that understanding peripheral biochemical homeostasis will aid in developing diagnostic and theranostic tools to aid in disease management (Do, 2023). Moreover it is possible that breakdown in peripheral biochemical homeostasis could be why people with schizophrenia experience other medical conditions at a higher rate than in people without a psychiatric condition (Smith et al., 2013). Medical conditions that have a higher occurrence in people with schizophrenia include viral hepatitis, epilepsy, dyspepsia, liver disease, diabetes, blindness, thyroid disorders, coronary heart disease, pain, psoriasis and eczema, chronic obstructive pulmonary disease, migraine and hearing loss. It has been suggested that people with schizophrenia may have problems with untreated medical conditions due to poorer access to medical services (Carney et al., 2006) or due to unwanted side effects of antipsychotic drug treatment (Mazereel et al., 2020). However, with the recognition that the breakdown of biochemical homeostasis in people with schizophrenia extends beyond the CNS (Scarr et al., 2015; Lai et al., 2016a) some consideration should be given as to whether CHRM1 / CHRM4-mediated biochemical abnormalities in people with the disorder could extend beyond the CNS.

In considering potential roles of the CHRM1 and CHRM4 in comorbid central and peripheral disorders, it is significant that mutations in the CHRM1 may be involved in the genesis of epilepsy (Marcé-Grau et al., 2021). In relation to the systemic illnesses more prevalent in people with schizophrenia, recent reports of the presence of CHRM1 on the pancreas (Pacher et al., 2019) and as a suggested drug target for the treatment of gastrointestinal and liver diseases (Tolaymat et al., 2021) are of interest. With regards to pain and schizophrenia, it is interesting that xanomeline has been shown to be an analgesic in the rodent models of inflammatory and neuropathic pain (Martino et al., 2011). In the case of higher rates of psoriasis and eczema, it is notable that the CHRM4 has been shown to have a role in modulating skin keratinocyte adhesion (Nguyen et al., 2004) which is important in skin repair. In the case of chronic obstructive pulmonary disease, it is significant that the CHRM1**^–^**^/^**^–^** mouse has changes in bronchoconstriction (Struckmann et al., 2003) and a pan-CHRM1, 2 and 3 antagonist is used to treat asthma (Matera et al., 2020). In addition, it has recently been reported that the CHRM4 in the pedunculopontine tegmental nucleus is a mediator of respiration in the rat (Lima et al., 2019). Finally, and somewhat surprisingly, the CHRM1**^–^**^/^**^–^** mouse has developmental abnormalities in the auditory cortex (Zhang et al., 2005) which raises the intriguing possibility that a deficit in CHRM1 may be involved in the increase in hearing loss in people with schizophrenia. Whilst all these observations currently remain a matter of conjecture, the possibility of abnormal CHRM signaling being involved in more than just the core symptoms of schizophrenia could be worthy of investigation.

## 3. Concluding remarks

The successful transition of the drug KarXT, a coformulation containing the CHRM1/CHRM4 agonist, through a large phase 2 trial (Brannan et al., 2021) and a phase 3 trial raises the prospect of a new treatment for schizophrenia that is not a dopamine D2 receptor antagonist that may also have some partial agonist properties. Moreover, early data from the trial of treating schizophrenia with emraclidine indicate selectively targeting the CHRM4 may also be a mechanism to reduce psychotic and negative symptom severity (Krystal et al., 2022). In addition, there are encouraging data showing the beneficial effects of using trace amine-associated receptor 1 (TAAR1) agonists to treat the symptoms of schizophrenia (Nair et al., 2022). It would therefore seem possible that drugs with different mechanisms of actions will be available for the treatment of schizophrenia and other disorders with similar symptoms. The completion of ongoing trials will hopefully provide more information on clinical benefits and side-effect profiles to help clinicians decide on more personalized treatment management plans.

**FIGURE 2:**
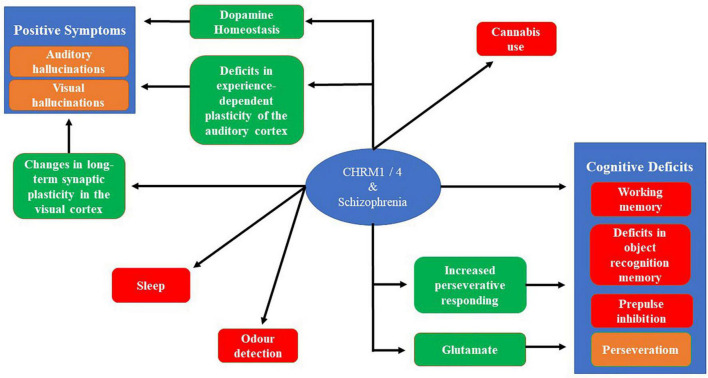
A schematic showing the connections between the CHRM1 and CHRM4 and the symptoms associated with schizophrenia. The red boxes show connections where there is a direct connection between and animal model and a symptom of schizophrenia. Brown boxes are symptom of schizophrenia that can be connect to CHRM1 and CHRM4 through an intermediate finding, shown in green boxes, using animal models or molecular studies. The absence of links to the negative symptoms reflects the absence of validated animal models and a poor understanding of the biological processes generating these symptoms.

Importantly, the discovery of the antipsychotic properties of chlorpromazine precede understanding the critical role of its action at the dopamine D2 receptor being critical to its antipsychotic effects (Shen, 1999). This contrasts to efforts to develop drugs to treat the symptoms of schizophrenia by targeting CHRMs because, as outlined in this review, evidence from different lines of research have helped explain why such an approach would have therapeutic benefits (Figure 2). Hence, if drugs targeting CHRMs to treat schizophrenia do enter clinical use this will not only be an exciting development for treating the disorder but would also argue for more intensive studies of its molecular pathology to identify potential new opportunities to develop new drug treatments. Such drugs will likely be necessary because, like in other areas of medicine, it is unlikely one drug will provide an optimum therapy for everyone within the syndrome of schizophrenia.

## Author contributions

BD prepared portions of the review and integrated individual contributions into a final version for review. GB and HU prepared specific sections of the review based on their specialist knowledge. RK provided the specialist clinical oversight. All authors contributed to the conceptualization of the scope of this review and involved in preparing the final manuscript submitted for publication.

## Footnotes

1 For ease of interpretation, in this review the abbreviation for muscarinic receptors will be consistently capitalized rather than alternating to upper- and lower-case abbreviations normal when citing studies in species other than humans and will be used instead of the recognized protein abbreviations ending in mAChR.
