# Supplementary material for: A growing understanding of the role of muscarinic receptors in the molecular pathology and treatment of schizophrenia
Source: Front Cell Neurosci. 2023 Feb 22;17:1124333. doi: 10.3389/fncel.2023.1124333 (PMC9992992; doi:10.3389/fncel.2023.1124333)
Supplement: Supplementary file 1 [file Data_Sheet_1.docx]

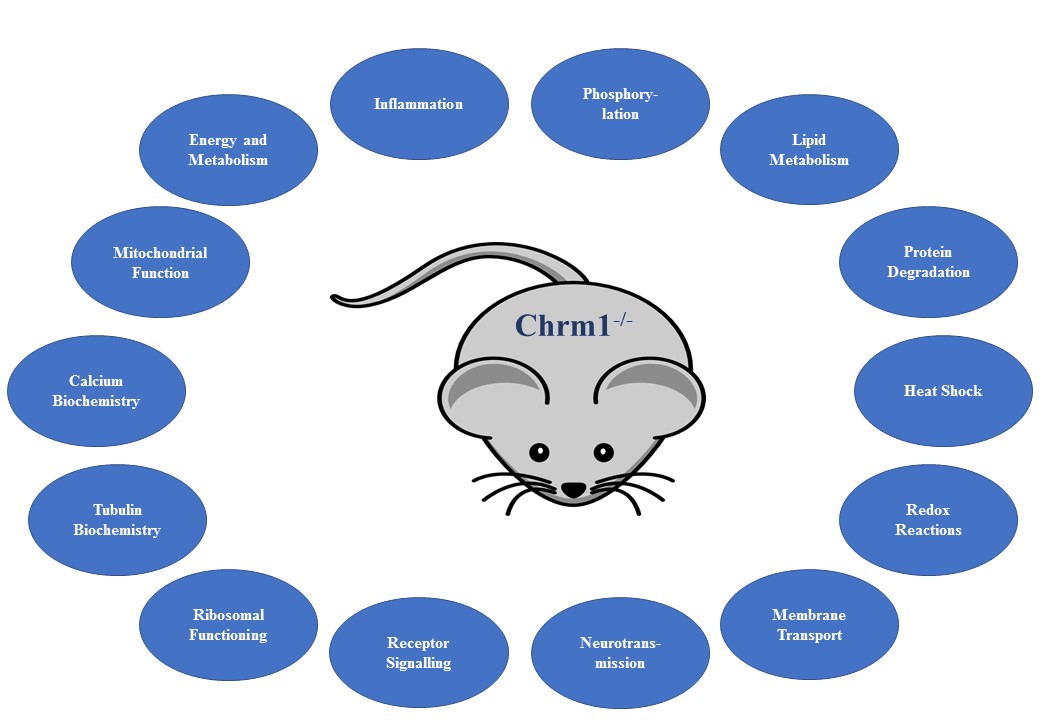


Supplementary Figure 1: A schematic showing the many pathways and functions predicted to be affected by changes in gene expression in the frontal cortex of the CHRM1^-/-^ mouse compared to the background strain wild type mouse.
